# Family‐level specialization in protein domain insertion architectures

**DOI:** 10.1002/pro.70586

**Published:** 2026-04-27

**Authors:** R. Dustin Schaeffer, Rui Guo, Jing Zhang, Qian Cong, Nick V. Grishin

**Affiliations:** ^1^ Department of Biophysics University of Texas Southwestern Medical Center Dallas Texas USA; ^2^ Eugene McDermott Center for Human Growth and Development University of Texas Southwestern Medical Center Dallas Texas USA; ^3^ Harold C. Simmons Comprehensive Cancer Center University of Texas Southwestern Medical Center Dallas Texas USA; ^4^ Department of Biochemistry University of Texas Southwestern Medical Center Dallas Texas USA

**Keywords:** domain architecture, domain classification, domain insertion, sequence families, structure prediction

## Abstract

Domain insertion creates architectures where one domain interrupts another's sequence. Analysis across 2.7 million classified domains reveals that insertions occur in 20% of multidomain proteins, with 331 families exhibiting consistent architectural roles: 162 function exclusively as hosts, while 169 exclusively serve as inserted modules, such as zinc‐binding dehydrogenases appearing as insertions across 450 events. The remaining 1116 families with sufficient insertion activity demonstrate versatile behavior, adopting different roles depending on partnership context. Size analysis shows inserted domains are consistently smaller than their hosts (median 115 vs. 199 residues), with role‐consistent families exhibiting 1.7‐fold size differences. Insertions frequently involve domains from different structural superfamilies: 31,925 events (65.8% of total) occur between families from different H‐groups, such as P‐loop hydrolases with tRNA modification domains. While most insertions are simple single‐level architectures, insertion mechanisms can create complex organizations, including six‐level nested structures in cyanobacterial RNA polymerase. This work provides a comprehensive dataset of 48,551 insertion events across 5701 families, with quantitative characterization of size relationships and partnership patterns that can inform structure prediction and protein design efforts.

## 1 INTRODUCTION

Protein domains represent fundamental structural and functional units that can operate independently or in complex multidomain arrangements (Chothia & Gough, 2009; Lees et al., 2016; Medvedev et al., 2021). Understanding how multiple domains contribute to protein function remains a significant challenge in structural biology, particularly when function depends on coordination among domains rather than on the independent activities of individual domains.

Domain insertion patterns, in which one domain is embedded within the sequence span of another (A1‐B‐A2 architecture), provide a tractable model system for studying multidomain relationships. Characterized initially by Russell (1994) through analysis of five hand‐curated examples, domain insertion creates architectures in which the inserted domain cannot simply be appended to the existing structure but must be compatible with the interruption of the host domain's sequence continuity (Russell, 1994). This architectural constraint makes insertion patterns useful test cases for analyzing domain partnership and simple functional architectures.

Domain insertion should be distinguished from circular permutation, where N‐ and C‐termini relocate within a single domain without creating a discontinuous sequence arrangement that accommodates a second domain. While both phenomena alter the relationship between sequence and structure, only insertion architectures involve distinct structural units with the A1‐B‐A2 topology analyzed here. The two phenomena can co‐occur: inserted domains may themselves be circularly permuted relative to their standalone homologs, as observed in some RNAP insertional domains (Manriquez‐Sandoval & Fried, 2022). Our analysis focuses specifically on the insertion relationship between domain pairs rather than internal rearrangements within individual domains.

Domain insertion is a subset of a broader range of domain accretions, in which complexity arises from the combination of functional modules. Computational sequence analysis and phylogenetic analysis of RNA polymerase evolution have established that the accretion of distinct sets of domains is a mechanism for diversification (Iyer et al., 2003, 2004). Subsequent experimental structures have shown that lineage‐specific insertions are responsible for functional specialization while maintaining overall domain architecture (Chlenov et al., 2005).

Study of structural domain insertion has progressed through several phases over three decades. Aroul‐Selvam et al. (2004) conducted the most comprehensive previous analysis, identifying insertion patterns in 9% of unique domain architectures present in SCOP 1.6.1 (Andreeva et al., 2020; Aroul‐Selvam et al., 2004). More recently, Manriquez‐Sandoval and Fried (2022) developed DomainMapper to address the challenge that discontinuous domains pose for sequence‐based annotation, a difficulty reflecting both a conceptual tension between treating domains as independent units and the reality of insertion‐mediated interdependence, and a practical sampling problem: insertion architectures may be sufficiently rare that tools developed on earlier databases encode biases from eras when examples were sparse. Validating their approach across eight model organisms using ECOD, they reported that approximately 5% of human domains and up to 10% of domains in other organisms exhibit non‐contiguous topology.

Detailed structural analysis within superfamilies has revealed co‐evolutionary relationships between host and inserted domains (Pandya et al., 2013), where partners undergo coordinated structural changes that may constrain successful architectural combinations. However, analysis of which domain families consistently participate in these partnerships across the broader structural proteome has remained limited by database scale and methodological constraints. Computational approaches have revealed specific challenges associated with insertion architectures. Structure prediction methods face difficulties with domain insertions because the two linkers create interdependent degrees of freedom that complicate standard domain assembly approaches (Berrondo et al., 2008). Recent protein engineering applications have achieved improved success rates when training sets include natural insertion patterns, suggesting that understanding natural partnership relationships may inform design efforts (Wolf et al., 2025). Computational approaches to domain architecture analysis have diversified substantially. Deep learning methods including Merizo (Lau et al., 2023) and Chainsaw (Wells et al., 2024) provide structure‐based domain segmentation trained on CATH classifications, while SWORD addresses the inherent ambiguity in domain boundary definitions by enumerating alternative valid decompositions (Postic et al., 2017). DomainMapper (Manriquez‐Sandoval & Fried, 2022) applies ECOD classifications to identify non‐contiguous domain topologies, reporting that 1.72%–4.26% of domains exhibit insertional character depending on the organism. These methods address sequential stages of domain analysis: partition tools first identify and assign domains within structures, and our approach then characterizes the architectural relationships between the resulting classified domains across the proteome.

Domain insertion is relatively rare, observed in only 9% of unique domain architectures in the most recent previous analysis (Aroul‐Selvam et al., 2004). This rarity creates a fundamental challenge: analyzing family‐level domain insertions requires sufficient examples of each participating domain family. Although the transition from SCOP 1.61 (Andreeva et al., 2020) to PDB‐based versions of ECOD markedly increased the database size, it still yielded a protein database with a similar sampling bias. The breakthrough performance of AlphaFold at CASP14 (Jumper et al., 2021; Kinch et al., 2021) followed by the release of the AlphaFold Database (AFDB) significantly altered this constraint. Integration of these predictions into domain classifications, including CATH (Waman et al., 2025), ECOD (Schaeffer et al., 2025), and Pfam (Paysan‐Lafosse et al., 2025), provided a scale and sampling sufficiently different to revisit this analysis. Our recent integration of AFDB and PDB structures in ECOD established a dataset of 2.7 million classified domains (Paysan‐Lafosse et al., 2025; Schaeffer et al., 2025). After 70% sequence identity clustering, this dataset comprises 179,697 multidomain proteins, a 257‐fold increase over the previous analysis. This scale provides improved characterization of which domain families consistently function as hosts or insertions across the protein universe.

Here, we present an analysis of family‐level architectural roles in domain insertion within the ECOD classification framework. We detect insertion events in multidomain proteins, characterize family‐level behavior patterns, and analyze the physical and functional properties of families that adopt specific architectural roles. We define an insertion event to describe a structural observation, an A1‐B‐A2 pattern detected in a protein, rather than to imply a specific evolutionary occurrence. Each “event” represents one detected instance of domain insertion architecture in a non‐redundant dataset. We use “host” and “inserted” (or “insertion”) as strictly topological descriptors: the host domain exhibits discontinuous sequence topology (A1–A2) while the inserted domain (B) occupies the intervening sequence. These terms describe structural arrangement rather than functional primacy; an ‘inserted’ domain may provide the catalytically essential function while the “host” serves a structural role. The central question addressed is: Do domain families consistently exhibit similar roles in insertion architectures, as classified in ECOD? Our analysis reveals insertion events in 20.23% of multidomain proteins, involving 5701 unique ECOD families. Among families with sufficient insertion activity, 331 families exhibit consistent roles—either exclusively functioning as hosts or exclusively as inserted modules. Analysis of these role‐consistent families reveals size relationships that characterize family‐level insertion behavior. This work presents the first large‐scale characterization of family‐level architectural roles in domain insertion within ECOD, providing a comprehensive dataset of insertion events and characterized insertion architectures.

## 2 RESULTS AND DISCUSSION

### 2.1 Domain insertion prevalence and distribution

**FIGURE 1:**
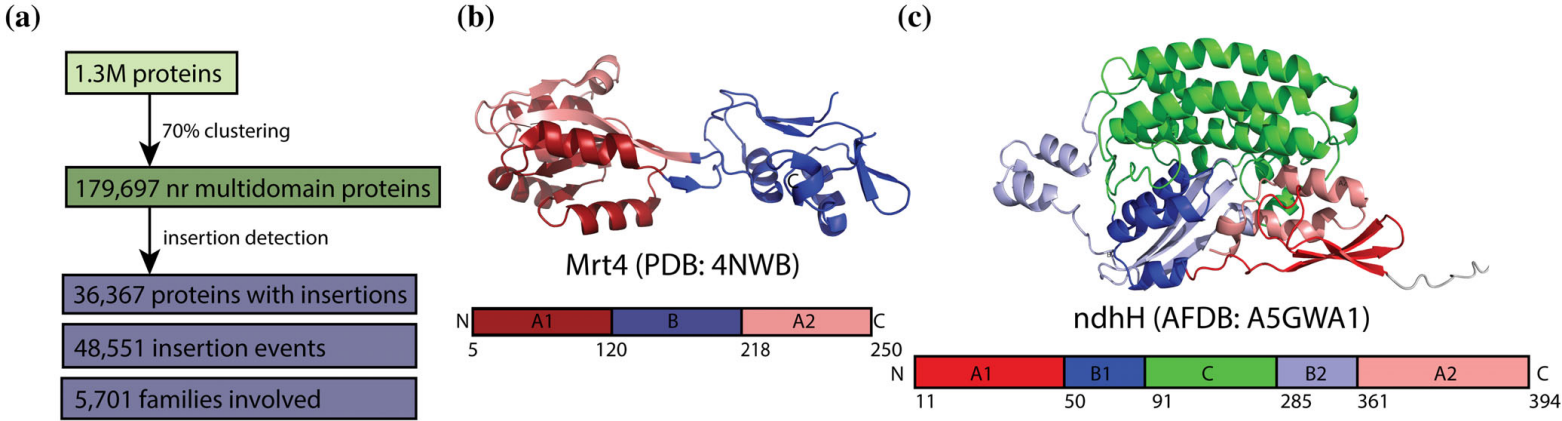
Insertion detection summary statistics and examples of simple and complex insertion cases. (a) Computational pipeline from ECOD database through clustering and insertion detection. (b) Simple domain insertion architecture in mRNA turnover protein 4 (PDB: 4NWB) showing A1‐B‐A2 pattern where domain B (blue) interrupts domain A (red). Sequence interruption does not prevent proper domain folding. (c) Nested insertion architecture in NADPH quinone oxidoreductase subunit ndhH (AlphaFold: A5GWA1) showing hierarchical A1‐B1‐C‐B2‐A2 pattern. This generates two independent insertion events: B→A and C→B.

To examine whether domain families exhibit consistent roles in insertion partnerships, we first characterized the overall landscape of insertion events within ECOD (Figure 1a). A1‐B‐A2 insertion detection across the 179,697 multidomain protein dataset reveals that domain insertion architectures are prevalent throughout the structural proteome. Domain insertions were identified in 36,367 proteins, yielding an insertion prevalence of 20.23% among multidomain proteins. The mRNA turnover protein Mrt4 (PDB: 4NWB) demonstrates typical A1‐B‐A2 architecture where domain sequence interruption does not prevent proper domain folding (Leidig et al., 2014) (Figure 1b). While most insertion events involve simple architectures, some proteins exhibit remarkable complexity, as exemplified by the NADPH quinone oxidoreductase subunit ndhH (AFDB: A5GWA1), which contains nested A1‐B1‐C‐B2‐A2 patterns that generate multiple independent insertion events (Figure 1c) (Varadi et al., 2024). Insertion‐containing proteins harbor 128,610 total domains, with an average of 3.54 domains per protein, demonstrating a correlation between insertion architecture and increased domain complexity. The analysis detected 48,551 individual insertion events spanning 5701 unique ECOD families, representing 28.7% of all classified families (19,844 total) and providing sufficient diversity for family‐level partnership analysis. These findings establish that domain insertion represents a widespread strategy across the structural proteome, occurring in approximately one‐fifth of multidomain proteins and involving over a quarter of all classified domain families. The detection of 48,551 insertion events across.

5701 families provide a sufficient statistical foundation for examining our central question: whether domain families exhibit consistent roles in insertion partnerships?

### 2.2 Family‐level partnership patterns

**FIGURE 2:**
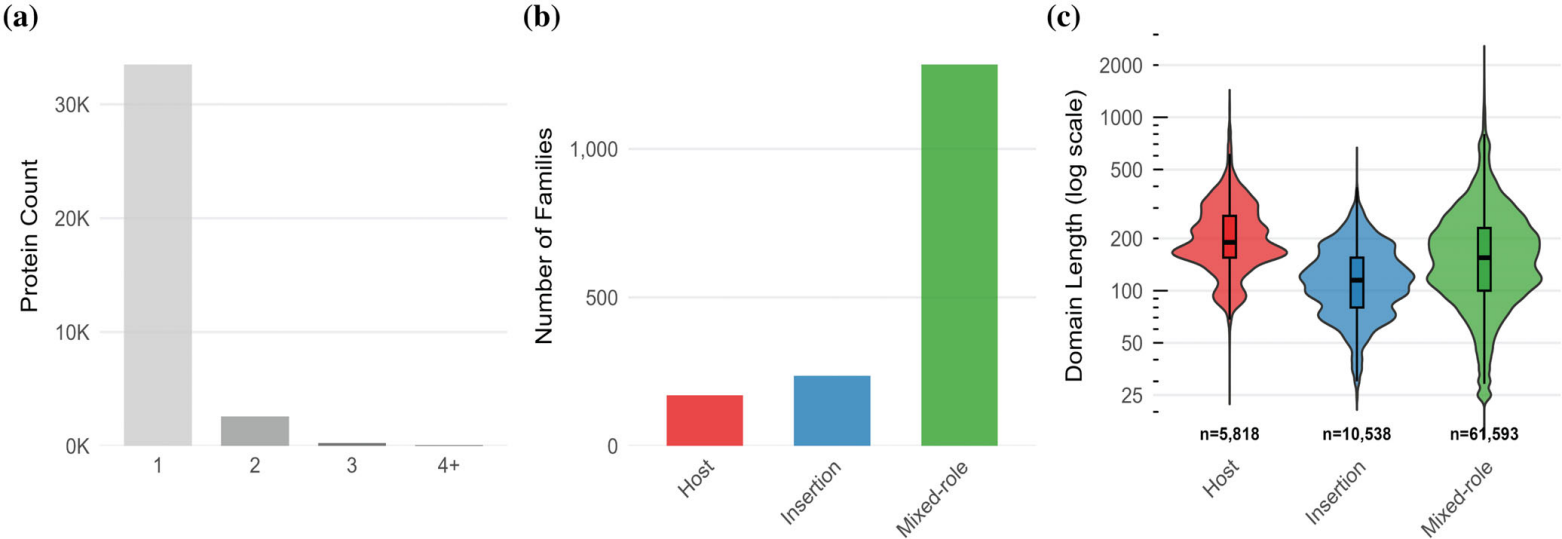
Systematic analysis of domain insertion partnerships reveals architectural hierarchy and family‐level specialization patterns. (a) Distribution of maximum nesting depth among insertion‐containing proteins shows that simple single‐level architectures dominate, while hierarchical complexity through recursive nesting patterns occurs in a minority of proteins. (b) Classification of domain families by partnership role consistency demonstrates systematic specialization into host families, insertion families, and mixed‐role families among families with sufficient insertion activity. (c) Domain length distributions for families with different partnership roles reveal systematic size relationships between host and insertion domains on a log scale.

To investigate whether families function exclusively as hosts, exclusively as insertions, or adopt versatile roles, we analyzed the partnership behavior of the 5701 families observed in insertion events. Role classification identified 331 families exhibiting strict role consistency across all observed insertion partnerships (Figure 2b). Of these, 162 families are host‐consistent, appearing only in the host position. 169 families are insertion‐consistent, appearing only as insertions. The remaining 1116 families demonstrate versatile behavior, participating in both roles. The role‐consistent families represent 22.9% of the 1447 families with sufficient insertion activity (≥10 events) for reliable assessment. Host‐consistent families tolerate sequence interruption across diverse biological functions, while insertion‐consistent families function as portable modules that integrate into existing domain architectures.

Size analysis across 48,263 paired domain measurements (Table S1) demonstrates that inserted domains are smaller than their hosts, with median lengths of 115 residues for insertions compared to 199 residues for hosts (Wilcoxon signed‐rank test, *p* <2.2e‐16). This constraint appears in 77.0% of insertion events, indicating that architectural compatibility requires size limitations for successful integration.

These size constraints reflect multiple structural requirements beyond compactness. Domain insertion architectures raise questions about folding order: the host domain's sequence is interrupted, potentially requiring either sequential folding (where the inserted domain completes first) or coordinated cofolding (Berrondo et al., 2008). The preference for smaller inserted domains may facilitate this folding hierarchy by ensuring inserted domains can achieve stable conformations before host domain closure.

Beyond size, N/C‐terminal proximity represents an independent geometric constraint on inserted domains. Analysis of Cα distances between the first and last residues of domain structures reveals that inserted domains have substantially closer termini than background domains (median 14.3 Å vs. 29.8 Å, Mann–Whitney U rank‐biserial *r* = 0.52) (Figure S2). This effect persists after controlling for domain size: within every size bin from 50 to 500+ residues, inserted domains maintain significantly shorter termini distances (*r* = 0.23–0.56). This constraint follows directly from the A1‐B‐A2 topology: the inserted domain must connect to both sides of the host gap, and distant termini would require long, potentially destabilizing linkers. Host domains, by contrast, show termini distances indistinguishable from background (*r* = −0.065), consistent with the requirement being specific to the insert role.

Analysis at the ECOD possible homology (X‐group) level identifies topologies that are structurally incompatible with insertion. Of 2017 X‐groups with sufficient representation, 421 are significantly depleted as inserts (Fischer's exact test, FDR <0.05), and 1054 have zero observed insert instances. The most strongly excluded topologies include ribosomal proteins, retroviral capsid proteins, and nuclear receptor ligand‐binding domains. Conversely, the most enriched insert topologies include domains whose names reflect their insertion function: tRNA IPPT transferase helical insertion domains (log2 enrichment = 5.3), XPD arch domains (4.4), and helicase helical bundle insertions (3.9). Depleted X‐groups have larger median termini distances than enriched X‐groups (weighted mean 29.8 Å vs. 21.7 Å; Spearman *ρ* = −0.44 between enrichment and termini distance, *p* = 9.6 × 10^−35^). However, termini proximity is necessary but not sufficient: several thousand protein families have close termini (e.g., L30p/L7e, 12.2 Å) yet never appear as inserts, likely because they cannot fold or function independently outside the ribosome complex. These results suggest three requirements for a domain to function as an insert: compact size (typically 50–200 residues), close N/C termini (~10–15 Å), and the capacity for folding independent of the host context.

Analysis of insertion positions within host domains reveals a uniform distribution: 32% N‐terminal, 34% middle, and 34% C‐terminal thirds (mean position = 0.507), indicating broad structural tolerance for sequence discontinuity rather than positional constraints on insertion sites. This uniformity suggests that size and termini geometry, rather than specific host domain locations, represent the primary determinants of successful insertion architecture.

Family‐level analysis reinforces role‐consistent patterns: insertion‐consistent families average 133.4 residues, compared to 241.2 residues for host‐consistent families, a 1.8‐fold difference in size. This size constraint extends to versatile families: among 2632 families that participate in both roles, domain instances average 78 residues larger when functioning as hosts compared to when the same families serve as insertions, indicating that size constraints operate within families rather than merely distinguishing between them. Most proteins contain simple single‐level insertions, with hierarchical nesting occurring in a minority of cases (Figure 2a). Domain‐length distributions show systematic size differences across families with different partnership roles (Figure 2c), demonstrating family‐level specialization. Analysis across ECOD's hierarchical structure reveals that architectural specialization occurs primarily at the family level rather than the homologous group level: among 103 H‐groups with sufficient insertion activity, 36.9% contain exclusively versatile families, indicating that homologous group membership does not constrain partnership behavior.

Within structurally versatile H‐groups, individual families demonstrate strong role specialization. The glyceraldehyde‐3‐phosphate dehydrogenase‐like domains (ECOD 298.1) show insertion bias, with 9 of 13 families exhibiting obligate insertion behavior. The P‐loop hydrolase domains (ECOD 2004.1) contain 55 families distributed across all role categories, including both obligate host specialists and versatile families. This family‐level specialization within versatile homologous groups indicates that successful insertion partnerships depend on family‐specific evolutionary adaptations rather than broad structural constraints alone.

Cross‐H‐group partnerships represent 31,925 insertion events (65.8% of total) between families from evolutionarily distinct structural categories, demonstrating that insertion architectures bridge independent evolutionary lineages. These relationships indicate that family‐level partnership preferences extend across the hierarchical classification structure rather than being confined to structurally related families.

Role‐consistent families span diverse biological functions. The five most frequently observed host‐consistent families (Table S2) include IPPT transferase (ECOD 2004.1.1.71, PF01715, 312 insertion events), dihydrodipicolinate reductase N‐terminus (ECOD 2003.1.1.12, PF01113, 193 events), Sec23/Sec24 beta‐sandwich domain (ECOD 11.1.5.29, PF08033, 174 events), E1‐E2 ATPase (ECOD 5073.1.2.1, PF00122, 128 events), and FtsX‐like permease (ECOD 1075.1.2.1, PF02687, 124 events). Conversely, the five most active insertion‐consistent families include zinc‐binding dehydrogenase (ECOD 2003.1.1.5, PF00107, 450 events), IPP transferase (ECOD 3949.1.1.1, PF01715, 283 events), leucyl‐tRNA synthetase editing domain (ECOD 1.1.1.7, PF13603, 213 events), dihydrodipicolinate reductase C‐terminus (ECOD 298.1.1.9, PF05173, 169 events), and glycosyltransferase family 28 C‐terminal domain (ECOD 7512.1.1.12, PF04101, 163 events).

The most frequent cross‐H‐group partnership linking P‐loop hydrolase family 2004.1.1.71 with tRNA modification family 3949.1.1.1 across 280 insertion events. These preferences align with structural studies showing co‐evolutionary coupling between insertion partners (Pandya et al., 2013), suggesting that successful domain combinations require not only architectural compatibility but also ongoing evolutionary coordination between partner families.

Insertion‐consistent families demonstrate that certain domains function as portable modules across diverse structural contexts. Zinc‐binding dehydrogenases (ECOD 2003.1.1.5), for example, appear as insertions in 450 events, with 98.9% specificity for GroES‐related hosts. In contrast, leucyl‐tRNA synthetase editing domains (ECOD 1.1.1.7) insert exclusively into tRNA synthetase hosts across 213 events, reflecting functional coupling between catalytic and editing activities within a single polypeptide. The strict insertion behavior of these families, zero instances of host function across hundreds of events, indicates that architectural role can be as conserved as sequence or structure. Whether this conservation reflects intrinsic structural features (e.g., compact geometry, termini orientation) or evolutionary contingency (e.g., founder effects in early protein evolution) remains an open question.

Analysis of host partnership diversity reveals a spectrum from specialists to generalists among insertion families. Among 581 insertion families with sufficient activity (≥10 events), 41.1% are strict specialists that partner with only a single host H‐group, while 2.4% are true generalists appearing in 11 or more distinct host superfamilies. The specialists include the zinc‐binding dehydrogenases and tRNA modification domains. In contrast, the generalist inserters are predominantly signaling and regulatory domains: PX domains (ECOD 277.1.1.1, PF00787; 21 host H‐groups), RING zinc fingers (ECOD 376.1.1.21, PF13639; 21 host H‐groups), C2 domains (ECOD 11.2.1.1, PF00168; 17 host H‐groups), PH domains (ECOD 220.1.1.1, PF00169; 15 host H‐groups), and PDZ domains (ECOD 7.1.1.1, PF00595, 13 host H‐groups). These well‐characterized protein–protein interaction modules function as portable regulatory elements across diverse enzymatic and structural scaffolds. The bimodal distribution, with strong enrichment at both extremes, suggests that insertion families evolve toward either functional coupling with specific partners or broad architectural compatibility enabling promiscuous partnership.

Our finding that 20.23% of multidomain proteins contain insertion architectures complements domain‐level statistics reported by DomainMapper (Manriquez‐Sandoval & Fried, 2022), which identifies approximately 5% of human domains as exhibiting non‐contiguous topology. These statistics reflect different denominators rather than conflicting observations: DomainMapper reports the fraction of all domains that are non‐contiguous, while we report the fraction of multidomain proteins containing at least one insertion event. Because each insertion event involves multiple domains (minimally one host and one insert), protein‐level prevalence will exceed domain‐level prevalence. Both analyses use ECOD domain definitions, providing methodological consistency. Our fold‐specific enrichment patterns align with DomainMapper's observations regarding TIM barrels, aminoacyl‐tRNA synthetases, and small compact domains as preferred hosts and insertions, respectively. We extend these observations by characterizing family‐level role consistency and cross‐superfamily partnerships, identifying 331 families with consistent architectural rates.

### 2.3 Case studies of insertion partnerships

The insertion preferences identified above raise questions about the biological significance of specific domain combinations. Cross‐H‐group insertions provide compelling examples, with 31,925 events occurring between families from evolutionarily distinct structural categories.

**FIGURE 3:**
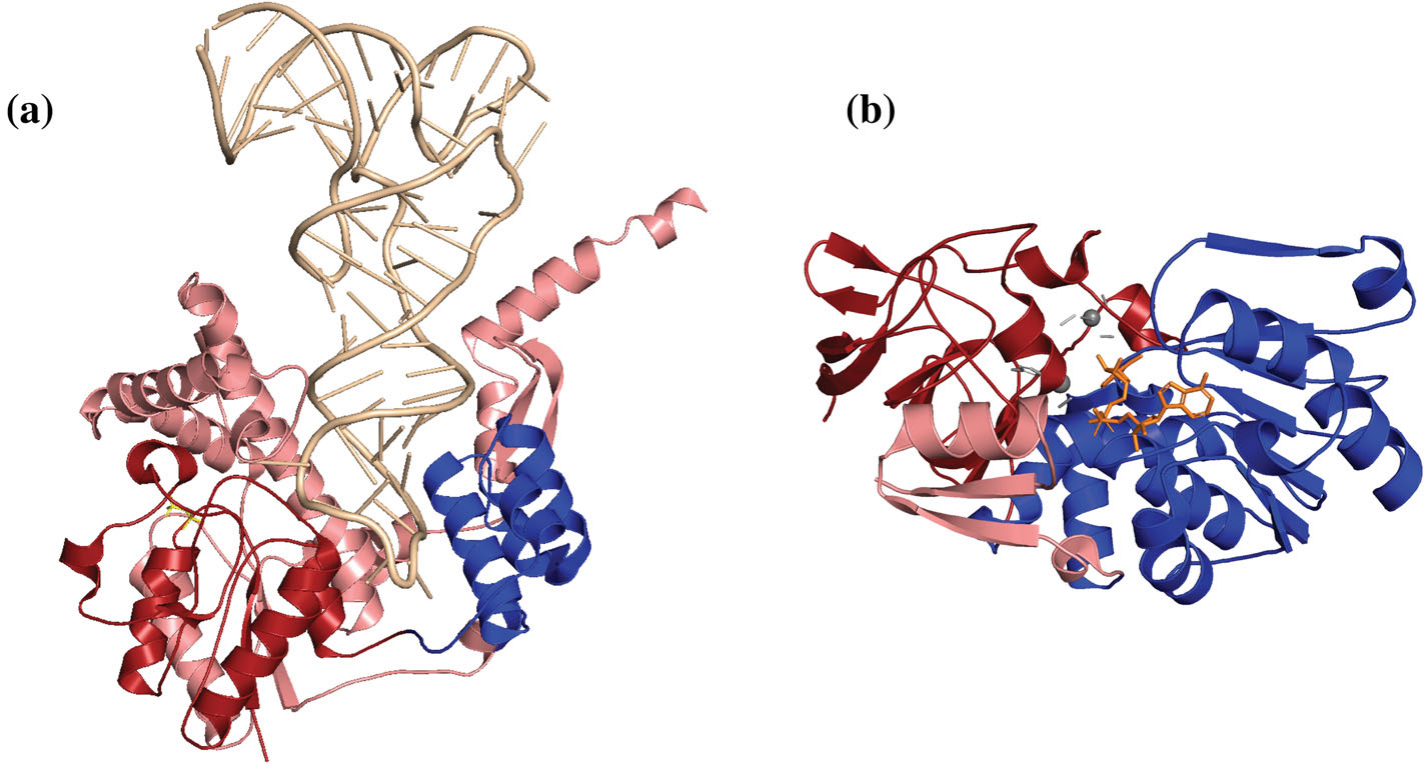
Cross‐H‐group insertion partnerships exemplifying domain cooperation. (a) IPPT transferase partnership in *Saccharomyces cerevisiae* MOD5 (PDB: 3EPL). P‐loop hydrolase host domain (red/salmon, residues 1–111 and 177–409) forms a discontinuous architecture around tRNA modification domain insertion (blue, residues 112–176). Bound tRNA (wheat) demonstrates coordinated substrate utilization between families 2004.1.1.71 and 3949.1.1.1 (280 partnership events, complete role specificity). (b) Zinc dehydrogenase partnership in mouse retinol dehydrogenase (PDB: 1E3I). GroES‐related host domain (red/salmon, residues 1–167 and 342–376) accommodates zinc dehydrogenase insertion (blue, residues 168–341) with coordinated NAD binding (orange) and zinc coordination (gray spheres). Partnership between families 236.1.1.1 and 2003.1.1.5 represents 450 events with 98.9% specificity.

The IPPT transferase partnership (ECOD 2004.1.1.71 and 3949.1.1.1) exemplifies cross‐H‐group role complementarity (Figure 3a). In the dimethylallyltransferase from *Saccharomyces cerevisiae* (PDB: 3EPL), the P‐loop hydrolase host accommodates the insertion of a tRNA modification domain, creating an integrated nucleotide‐binding and substrate modification architecture (Zhou & Huang, 2008). The P‐loop hydrolase family (ECOD 2004.1.1.71) functions exclusively as a host (312 host events, 0 insertions), and the tRNA modification family (ECOD 3949.1.1.1) appears exclusively as an insertion (283 instances across 280 distinct proteins). Three proteins contain two instances of this family as insertions within the same host domain architecture. This partnership links evolutionarily distinct structural categories: P‐loop ATPases (H‐group 2004.1) and tRNA modification domains (ECOD 3949.1), thereby creating dimethylallyltransferases with both nucleotide‐binding and tRNA modification domain architectures within a single polypeptide chain.

Zinc‐binding dehydrogenases (ECOD 2003.1.1.5) exhibit highly specific partnership patterns (Figure 3b). Mouse retinol dehydrogenase (PDB: 1E3I) exemplifies this partnership, with GroES‐related host domains providing NAD‐binding frameworks while zinc dehydrogenase insertions contribute metal coordination sites (Svensson et al., 2000). This partnership links GroES‐related hosts (ECOD 236.1) with Rossmann‐fold zinc dehydrogenases (ECOD 2003.1), creating alcohol dehydrogenase enzymes containing both NAD‐binding and zinc‐coordination architectures.

Analysis of ECOD family 1.1.1.7 (tRNA‐synt_1_2, PF13603) reveals leucyl‐tRNA synthetase editing domains as insertion‐exclusive modules: 213 insertion events with 100% insertion behavior and 100% of events involving tRNA synthetase hosts. The primary pairing involves tRNA synthetase domains (ECOD 2005.1.1.29, tRNA‐synt_1g, PF09334) as hosts for 125 insertion events (58.7%), with classic tRNA synthetase families (ECOD 2005.1.1.1 and 375.1.1.1, both PF00133) contributing 72 additional events and anticodon recognition domains (ECOD 140.1.1.5, PF08264) accommodating the remainder.

Size analysis across 48,263 paired domain measurements reveals constraints governing successful partnerships. Inserted domains are smaller than their hosts in 77.0% of events, with median lengths of 115 residues versus 199 residues for insertions and hosts, respectively (Wilcoxon signed‐rank test, *p* <2.2e‐16). Insertion‐consistent families average 133.4 residues compared to 241.2 residues for host‐consistent families (1.8‐fold difference), suggesting that compact, structurally compatible domains integrate more successfully into existing architectures. Analysis of insertion positions within host domains reveals a uniform distribution: 32% N‐terminal, 34% middle, and 34% C‐terminal thirds (mean position = 0.507), indicating broad structural tolerance for sequence discontinuity rather than localized insertion sites.

Analysis across ECOD's 20 architectures reveals structural preferences governing role selection (Table S3, Figure S1). We calculated enrichment by log odds ratio (log_2_OR) as described in Methods. Domains with few secondary structure elements (architecture a.19) show strong enrichment as insertions (log_2_OR = −1.62, representing a 3‐fold enrichment), as expected for compact folds within host domain gaps. Beta meanders (a.2, log_2_OR = −1.04) and alpha arrays (a.6, log_2_OR = −0.96) similarly favor insertion roles. Conversely, multi‐layer α + β architectures (a.12) are host‐enriched (log_2_OR = 1.24), suggesting that complex layered folds tolerate sequence discontinuity while providing stable scaffolds. Alpha superhelices and bundles (a.9, a.7) also favor host roles (log_2_OR = 0.53, 0.48, respectively), potentially reflecting the flexibility of repetitive helical structures to accommodate integration. Notably, Rossmann‐like *α*/*β* sandwiches (a.17), comprising the largest fraction of insertion events, show no role preference (log_2_OR = 0.16), demonstrating that architectural abundance does not predict architectural role.

Cyanobacterial RNA polymerases represent a unique evolutionary elaboration consistent with established principles of systematic domain accretion (Iyer et al., 2003, 2004). Unlike typical bacteria, where a single rpoC gene encodes the *β*′ subunit, cyanobacteria split this gene into rpoC1 and rpoC2, creating a six‐subunit enzyme architecture first characterized in *Nostoc commune* (Xie et al., 1989). The rpoC2 subunit contains a large SI3 domain of 630+ residues—over three times larger than the equivalent domain in *Escherichia coli*. Recent cryo‐EM structures confirmed the functional significance of this SI3 domain organization in transcription initiation through the formation of a stabilizing SI3‐σ arch interaction (Shen et al., 2023).

**FIGURE 4:**
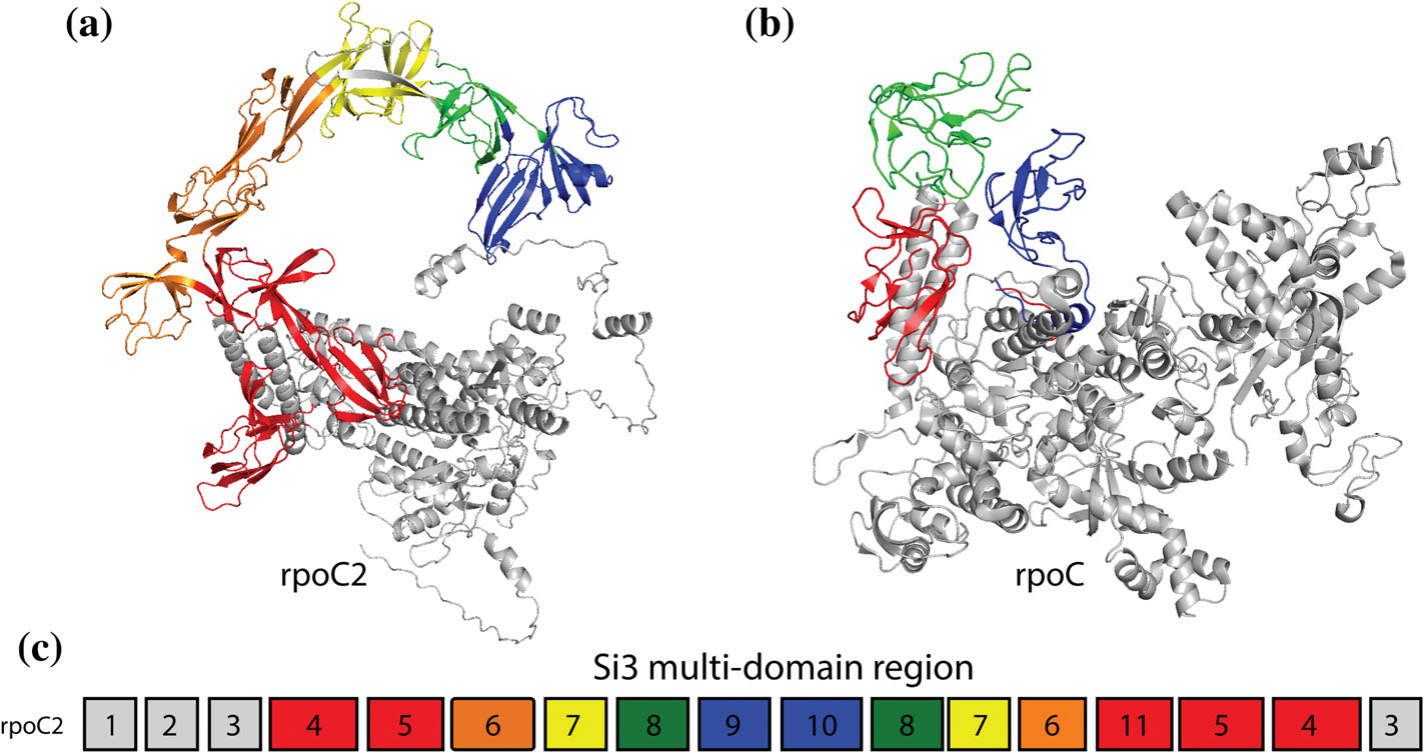
Complex hierarchical domain insertion architecture in RNA polymerase *β*′ subunits demonstrates ECOD's classification across organizational complexity levels. (a) AlphaFold structure of *Prochlorococcus marinus* rpoC2 (A2C6T1, 1280 residues) showing Si3 region with domains colored by insertion depth: Red (depth 1), orange (depth 2), yellow (depth 3), green (depth 4), blue (depth 5). Background host domains shown in gray. (b) *Escherichia coli* RNA polymerase *β*′ subunit (PDB: 6psu chain J, 1407 residues) showing baseline Si3 architecture (residues 932–1215, 284 aa) with three continuous domains colored sequentially (red, green, blue). Non‐Si3 regions in gray. Cyanobacterial rpoC1/rpoC2 gene split correlates with Si3 elaboration: Six cluster representatives show a 2.3–2.4× expansion (640–690 aa), with 8–10 domains and 5–7 internal insertion events relative to the *E. coli* baseline. (c) Linear domain organization schematic for A2C6T1 rpoC2 showing 12 sequential domains with Si3 region (domains 4–13) exhibiting hierarchical nesting architecture. Colors match structural representation in panel (a), demonstrating ECOD's classification of complex multidomain architectures through discontinuous domain definitions and insertion detection.

The AlphaFold structure of *Prochlorococcus marinus* rpoC2 subunit (AFDB: A2C6T1) exemplifies this complexity, with the Si3 multidomain region (residues 463–1007) containing eight domains organized in a 6‐level recursive insertion architecture (Figure 4a). In contrast, the *E. coli* rpoC protein (PDB: 6PSU, J) contains only three linear hybrid motif domains (ECOD 325.1.7) with no insertion architecture (Figure 4b). Analysis reveals that 33 of 59 insertions (55.9%) involve single hybrid motifs, which correspond to the HABAS family (CATH 2.40.50.100) recently characterized as a major insertional domain class in bacterial RNAP‐β’ subunits (Alvarez‐Carreño et al., 2024). The recursive pattern we observe is consistent with their description of Type 3 RNAP‐*β*′ architecture in cyanobacteria.

This recursive pattern creates hierarchical architectures of the form A1‐B1‐C‐B2‐A2, where domain B serves dual roles as both an inserted module (within domain A) and a host (for domain C). The nesting relationships reveal 12 total domains with bifurcating nodes where single hosts accommodate multiple insertions (Figure 4c), demonstrating that insertion architecture can achieve remarkable complexity through recursive application of the basic A1‐B‐A2 principle.

## 3 CONCLUSIONS

This analysis of domain insertion patterns across 2.7 million classified domains establishes that insertion events occur in 20.23% of multidomain proteins and involve 28.7% of all classified protein families. The identification of 331 families with consistent partnership roles provides the first comprehensive characterization of architectural specialization at the family level within ECOD. Cross‐H‐group partnerships (31,925 events) demonstrate systematic relationships between evolutionarily distinct structural categories. Physical property analysis reveals universal size constraints—insertions consistently smaller than hosts—operating across all partnership types. The capacity for hierarchical organization, demonstrated by recursive insertion patterns creating six‐level nested architectures in RNA polymerase, shows that these constraints enable rather than preclude architectural complexity. This characterization depends on ECOD's domain boundary definitions and inherits any biases present in the underlying structural data. While the integration of AlphaFold predictions proves unprecedented scale, whether this expansion fully escapes historical biases encoded in classification systems trained on earlier, smaller datasets remains an open question– insertion architectures sufficiently rare in earlier databases may remain underrepresented in the training data that shaped both prediction methods and classification frameworks. The observed consistency of partnerships—such as 100% insertion behavior in tRNA editing domains or 98.9% specificity in zinc dehydrogenase partnerships—could reflect genuine co‐evolutionary relationships or conserved structural features classified as separate families. Distinguishing these scenarios requires additional evolutionary and experimental evidence. The partnership patterns identified here provide a foundation for such investigations. The A1‐B‐A2 detection methodology demonstrates the feasibility of analyzing multidomain architectures at unprecedented scale, offering a pathway for characterizing complex protein organizational principles as structural databases expand.

## 4 METHODS

### 4.1 Database and data sources

The analysis used protein structure data from the ECOD (Evolutionary Classification of Protein Domains) database, which provides hierarchical domain classifications for experimentally determined and computationally predicted protein structures (Schaeffer et al., 2014, 2025). The complete dataset comprises 1,598,463 proteins sourced from multiple structural databases, including experimental structures from the Protein Data Bank and predicted structures from the AlphaFold Protein Structure Database (Burley et al., 2022; Varadi et al., 2024). Among these proteins, 659,312 contain multiple domains while 887,943 are classified as single‐domain proteins, yielding a multidomain rate of 41.2% across the complete dataset.

To reduce sequence redundancy while maintaining structural and functional diversity, the protein dataset was clustered using CD‐HIT at a 70% sequence identity threshold (Huang et al. 2010). The 70% sequence identity threshold represents a standard choice for reducing sequence redundancy while preserving functional and structural diversity. At this threshold, proteins within a cluster typically share the same domain architecture and functional annotation, while distinct clusters represent evolutionarily divergent sequences. Higher thresholds (e.g., 90%) would retain near‐duplicates that inflate event counts, while lower thresholds (e.g., 40%) would collapse functionally distinct proteins. The clustering process analyzed 1,302,599 input proteins and generated 405,078 non‐redundant cluster representatives, achieving an average cluster size of 3.22 proteins per cluster. The clustering identified 274,459 singleton clusters, each containing unique proteins, and 130,619 multi‐member clusters with sequence similarity above the threshold. The largest cluster contained 4135 proteins, indicating substantial sequence redundancy in certain protein families within the structural databases. Among the 405,078 cluster representatives, 377,713 (93.2%) had complete domain assignments from ECOD, comprising 179,697 multidomain proteins and 198,016 single‐domain proteins. The remaining 27,365 representatives (predominantly AlphaFold predictions) lacked domain assignments and were excluded from insertion analysis.

Among the 405,078 cluster representatives, 377,713 (93.2%) had complete domain assignments from ECOD, comprising 179,697 multidomain proteins (44.4%) and 198,016 single‐domain proteins (48.9%). The remaining 27,365 representatives (6.8%), predominantly AlphaFold predictions awaiting domain classification, were excluded from the insertion analysis. This multidomain enrichment relative to the complete dataset reflects the tendency of larger, more complex proteins to form singleton clusters during the redundancy reduction process. Domain boundary definitions and hierarchical classifications were obtained from ECOD v293, which incorporates complex interdomain geometries (Majumdar et al., 2009). The analysis incorporates both F‐group‐level classifications representing specific protein families and T‐group‐level classifications for domains that could not be assigned to established families. This classification framework enables systematic analysis of insertion patterns across the complete spectrum of known protein domain families while maintaining compatibility with existing structural biology databases and classification systems. H‐group‐level analysis examined whether architectural specialization occurs at the superfamily level. H‐groups containing at least three families with ≥10 insertion events were categorized as containing exclusively role‐consistent families, exclusively versatile families, or mixed behavior.

### 4.2 A1‐B‐A2 insertion event detection

Domain insertion events were identified by systematically examining multidomain proteins for patterns in which a domain appears split into two segments, with an intervening domain inserted between them. The algorithm analyzes the sequential arrangement of domains within each representative multidomain cluster. Domain family classifications were obtained from ECOD F‐group assignments, which include both specific F‐group classifications and T‐group‐only assignments (families terminating in “0.0”) for domains that could not be assigned to established families. While T‐group‐only families represent 5.3% of all classified domains in ECOD, they account for 25.2% of host domains and 32.7% of inserted domains in detected insertion events. For domains that share identical family classifications, the algorithm determines whether they represent segments of a single interrupted domain or independent domain repeats.

The detection process identifies gaps of at least 30 residues between consecutive segments of discontinuous domains, following established domain boundary threshold conventions in ECOD. The 30‐residue threshold reflects the minimum size observed for structurally independent domains: although smaller domains exist (e.g., metallothioneins, some zinc fingers), smaller regions typically lack sufficient secondary structure elements to form stable folds with a hydrophobic core. This threshold aligns with DomainMapper's “intra‐gap parameter” (Manriquez‐Sandoval & Fried, 2022) and represents a conservative estimate ensuring that detected gaps could plausibly accommodate folded domains rather than extended loops or linker regions. For each potential inserted domain, the algorithm evaluates all gaps in which the domain's sequence range falls entirely within the gap boundaries. To prevent double‐counting in proteins with multiple potential insertion sites, each domain is assigned exclusively to the smallest gap it completely occupies. For example, consider a protein with host domain A containing two nested insertions (i.e., A1‐B1‐C‐B2‐A2). Domain C is involved in an insertion event with domain B, but not domain A. This assignment strategy ensures each domain participates in at most one insertion event per protein.

Nested insertion architectures, in which inserted domains themselves serve as hosts for additional insertions, were identified through the recursive application of the insertion detection algorithm and are presented as illustrative examples (Figure 2a).

### 4.3 Family role classification

Domain families were systematically classified by their behavioral patterns during insertion events to identify families with consistent architectural roles. The classification process aggregates all insertion events for each ECOD F‐group family and calculates the total number of events in which each family functions as a host domain versus an inserted domain. This aggregation encompasses insertion events in which both the host and the inserted domains received F‐group classifications, as well as additional events involving T‐group classifications.

To ensure statistical robustness and eliminate families with insufficient data for reliable role assessment, only families participating in 10 or more total insertion events were included in the role consistency classification analysis. This threshold retained 1447 families for role assessment, representing 25.4% of all 5701 families participating in insertion events. Among these families meeting the activity threshold, 331 exhibited role consistency (22.9%), while 1116 families (77.1%) demonstrated versatile behavior across both host and insertion roles.

The classification system assigns families to one of three behavioral categories based on their participation patterns in insertion events. Host‐consistent domains demonstrate exclusive participation as host domains across all observed insertion events, exhibiting zero instances of functioning as inserted domains. Insertion‐consistent domains display the opposite pattern, participating exclusively as inserted domains, with no observed cases of host behavior. Versatile families participate in both host and insertion roles, indicating architectural flexibility rather than consistent partnership function. Family counts reported throughout this manuscript (5701 unique families) represent distinct ECOD F‐group classifications participating in insertion events. While some families function in both host and insertion roles, each family is counted only once. Alternative counting approaches that enumerate host‐role and insertion‐role instances separately would yield higher counts (7836 family‐role instances), but we report unique family participation to avoid double‐counting versatile families.

### 4.4 Physical property analysis of insertion families

Domain‐level size analysis was performed using sequence length measurements for all domains participating in insertion events (Table S1). For each A1‐B‐A2 insertion event, the lengths of the host domain (A1 + A2 combined) and inserted domain (B) were recorded, creating 48,263 paired measurements for statistical comparison.

The statistical significance of size differences was assessed using the Wilcoxon signed‐rank test for paired domain comparisons (host vs. insertion within each architectural event). This nonparametric paired test accounts for the relationship between host and insertion domains within the same protein while making no assumptions about the distribution of the data. Family‐level size characteristics were calculated by aggregating all domains within each F‐group family and computing the mean domain length for obligate host families, obligate insertion families, and versatile families. Size distribution analysis examined the frequency of cases in which inserted domains are smaller than their hosts, calculated size ratios (insertion length divided by host length), and determined the median absolute size difference. No size filtering criteria were applied to maintain the complete dataset for analysis.

### 4.5 Family ranking and partnership analysis

Family ranking was performed by counting the total number of insertion events for each F‐group family in their respective roles (Table S2). Host‐consistent families were ranked by the total number of insertion events in which they functioned as host domains, while insertion‐consistent families were ranked by the total number of events in which they served as inserted domains. Rankings presented in Results represent families with the highest event frequencies within each category. Cross‐H‐group partnerships were identified by analyzing insertion events that connect F‐group families belonging to different H‐groups (homologous superfamilies). Each A1‐B‐A2 insertion event linking families from different H‐groups was counted as a partnership instance between those H‐groups. Partnership quantification calculated the total number of insertion events connecting specific family pairs across H‐group boundaries. For example, the 283 events linking P‐loop hydrolase family 2004.1.1.71 with tRNA modification family 3949.1.1.1 represent a partnership between H‐groups 2004.1 and 3949.1. Systematic relationship identification focused on recurring partnerships that appear multiple times in the dataset rather than isolated occurrences. The top family selection for presentation in Results was based on raw event counts, showing the five highest‐ranking families in each role‐consistency category, along with their total event counts and, where available, associated Pfam family identifiers.

### 4.6 Software

Statistical analyses and data manipulation were performed using R version 4.4.1 (R Core Team, 2021) with the dplyr (Wickham et al., 2025) and ggplot2 (Wickham, 2016) packages. Multi‐panel figures were created using patchwork (Pedersen, 2025). Protein structure figures were generated using PyMOL version 3.0.4 (Schrodinger LLC, n.d.).

### 4.7 Use of AI‐assisted tools

Claude Code (Opus 4.6 and Sonnet 4.6) was used to assist with data analysis scripting, statistical computations, and revision preparation. All AI‐generated code and text were reviewed, validated, and edited by the authors. The authors take full responsibility for the content of this manuscript.

## AUTHOR CONTRIBUTIONS

**Rui Guo:** Writing – review and editing. **Nick V. Grishin:** Conceptualization; supervision; resources; funding acquisition; project administration; writing – review and editing. **Qian Cong:** Supervision; resources; funding acquisition. **R. Dustin Schaeffer:** Conceptualization, supervision, resources, funding acquisition; writing ‐ review and editing. **Jing Zhang:** Conceptualization, data generation.

## CONFLICT OF INTEREST STATEMENT

The authors declare no conflicts of interest.

## Supporting information


**Figure S1.** Architecture‐level enrichment in insertion roles. Horizontal bar chart showing log_2_ odds ratios for each ECOD architecture category, where positive values indicate host‐enrichment and negative values indicate insertion‐enrichment. Dashed lines mark ±0.5 threshold for classification. Colors: green = host‐enriched (>0.5), gray = neutral, red = insertion‐enriched (<−0.5).

**Figure S2.** Structural determinants of domain insertion. (A) Density distributions of C*α* distance between N‐ and C‐terminal residues for domains observed as inserts (red, *n* = 48,133), hosts (green, *n* = 38,025), and a random background sample (gray, *n* = 45,570). Dashed lines indicate group medians. Inserted domains have significantly closer termini (median 14.3 Å vs. ~30 Å; Mann–Whitney U rank‐biserial *r* = 0.52), a constraint that persists after controlling for domain size. (B) Top 20 ECOD X‐groups most significantly depleted as inserted domains (Fisher's exact test, FDR< 0.05), with domain counts in parentheses. Ribosomal proteins dominate, reflecting their obligate role in multisubunit complexes that preclude independent function as inserted modules.

**Table S1:** Domain size measurements.

**Table S2:** Insertion events and family classifications.

**Table S3:** Architecture enrichment analysis (table_S3.csv).

## Data Availability

The complete dataset of 48,551 insertion events, including domain size measurements (Table S1) and family classifications (Table S2), is available at the Zenodo repository: https://doi.org/10.5281/zenodo.17260165. All data are provided in machine‐readable formats to facilitate further analysis and structure prediction efforts.
