# Supplementary figures and images for: Family‐level specialization in protein domain insertion architectures
Source: Protein Sci. 2026 Apr 27;35(5):e70586. doi: 10.1002/pro.70586 (PMC13114784; doi:10.1002/pro.70586)

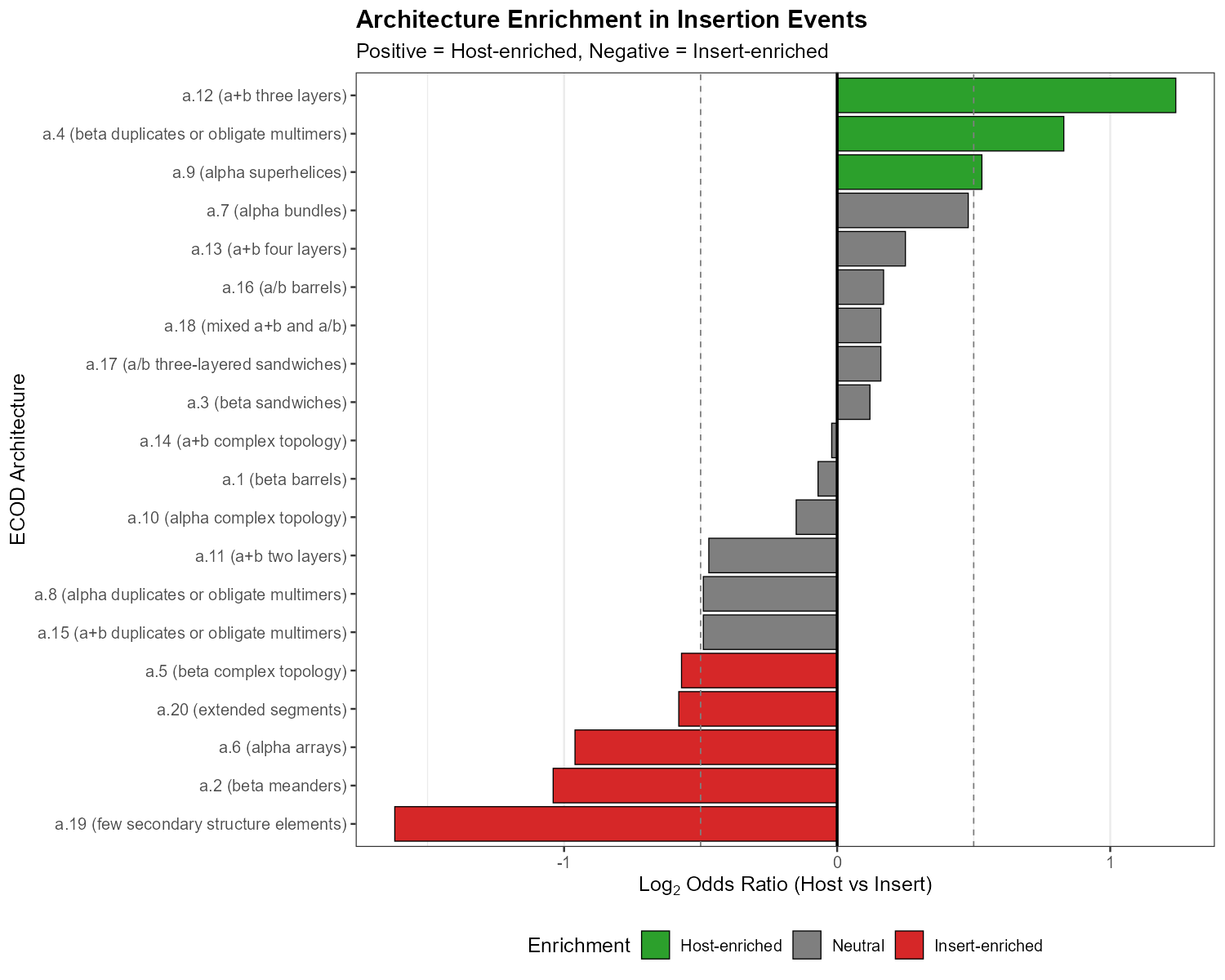

Supplement: Supplementary file 1 — Figure S1. Architecture‐level enrichment in insertion roles. Horizontal bar chart showing log2 odds ratios for each ECOD architecture category, where positive values indicate host‐enrichment and negative values indicate insertion‐enrichment. Dashed lines mark ±0.5 threshold for classification. Colors: green = host‐enriched (>0.5), gray = neutral, red = insertion‐enriched (<−0.5). [file PRO-35-e70586-s004.png]

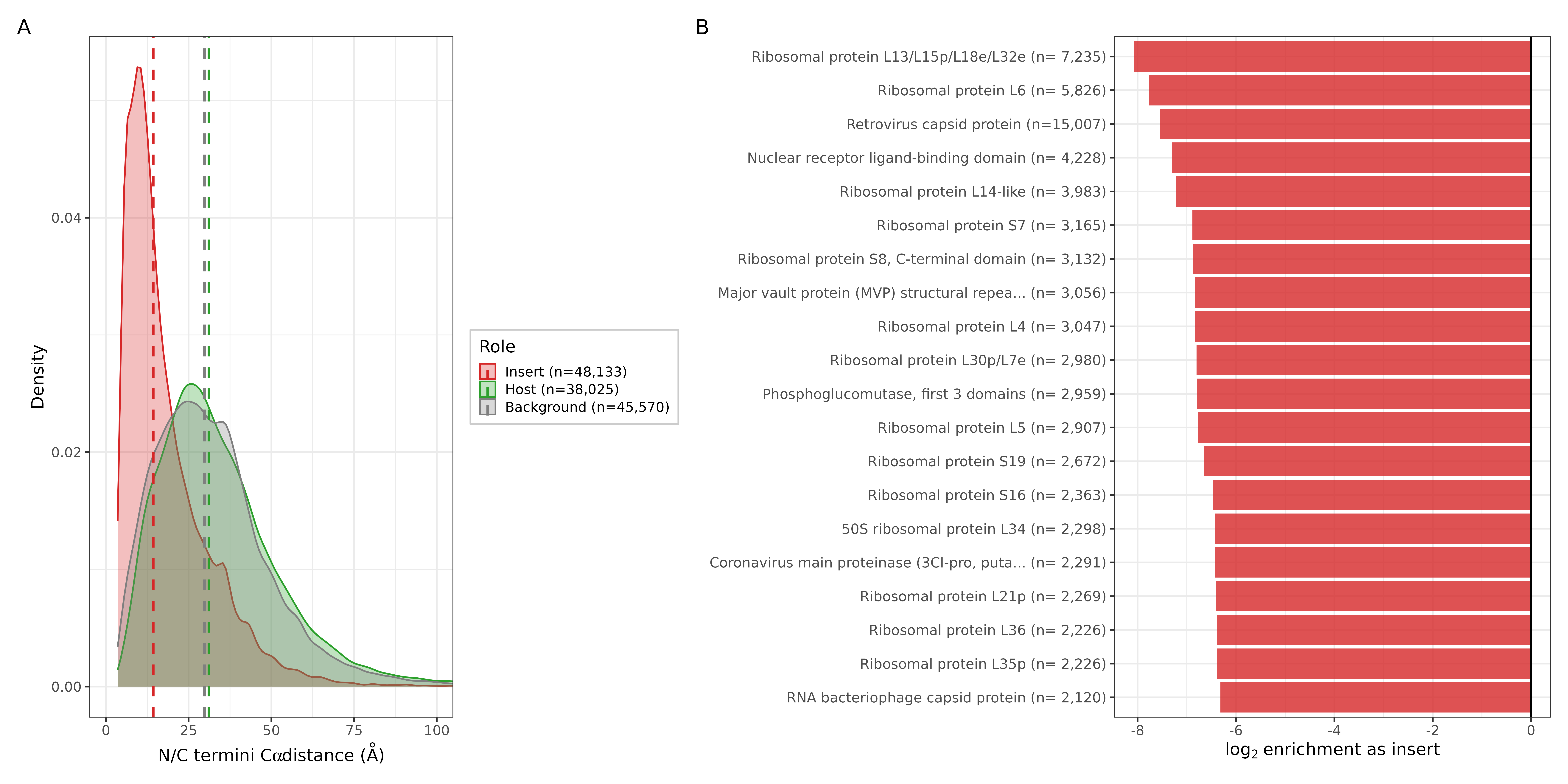

Supplement: Supplementary file 2 — Figure S2. Structural determinants of domain insertion. (A) Density distributions of Cα distance between N‐ and C‐terminal residues for domains observed as inserts (red, n = 48,133), hosts (green, n = 38,025), and a random background sample (gray, n = 45,570). Dashed lines indicate group medians. Inserted domains have significantly closer termini (median 14.3 Å vs. ~30 Å; Mann–Whitney U rank‐biserial r = 0.52), a constraint that persists after controlling for domain size. (B) Top 20 ECOD X‐groups most significantly depleted as inserted domains (Fisher's exact test, FDR< 0.05), with domain counts in parentheses. Ribosomal proteins dominate, reflecting their obligate role in multisubunit complexes that preclude independent function as inserted modules. [file PRO-35-e70586-s001.tiff]
